# Recurrent excitation between motoneurones propagates across segments and is purely glutamatergic

**DOI:** 10.1371/journal.pbio.2003586

**Published:** 2018-03-14

**Authors:** Gardave S. Bhumbra, Marco Beato

**Affiliations:** Department of Neuroscience, Physiology and Pharmacology, University College London, London, United Kingdom; Stanford University, United States of America

## Abstract

Spinal motoneurones (Mns) constitute the final output for the execution of motor tasks. In addition to innervating muscles, Mns project excitatory collateral connections to Renshaw cells (RCs) and other Mns, but the latter have received little attention. We show that Mns receive strong synaptic input from other Mns throughout development and into maturity, with fast-type Mns systematically receiving greater recurrent excitation than slow-type Mns. Optical recordings show that activation of Mns in one spinal segment can propagate to adjacent segments even in the presence of intact recurrent inhibition. While it is known that transmission at the neuromuscular junction is purely cholinergic and RCs are excited through both acetylcholine and glutamate receptors, here we show that neurotransmission between Mns is purely glutamatergic, indicating that synaptic transmission systems are differentiated at different postsynaptic targets of Mns.

## Author summary

Motoneurones (Mns) are the last elements of the networks that command, coordinate, and actuate the most essential of behaviours: movement. Their activation triggers muscle contractions, and the many diseases affecting Mns cause progressive and fatal paralysis. We show here that Mns themselves form an interconnected network and that activity in one segment of the spinal cord can propagate reciprocally to neighbouring segments, thus constituting a positive feedback loop that can amplify the strength of motor output. Remarkably, while Mns excite muscles through release of the neurotransmitter acetylcholine, our data show that the synapses between Mns operate through glutamate, providing a rare example of differentiation of transmitter systems according to the postsynaptic targets.

## Introduction

Motoneurones (Mns) are the ultimate neural targets of effector commands issued from the central nervous system. Their activity is modulated by an intricate network of interneurones [1] that affect the spatial and temporal distribution of excitation to different motor pools [2]. Mns also receive direct inputs from supraspinal tracts and sensory afferents, and their outputs are not confined to the peripheral muscles but also include excitatory collateral terminals to Renshaw cells (RCs).

Early anatomical studies [3] have shown that Mn axon collaterals have large ramifications that invade the motor nucleus and may form synaptic contacts with other Mns. An early evidence of functional connectivity between Mns was found in the adult cat [4] but was attributed to the presence of gap junctions. The first proof of the existance of chemical synapses between Mns was found in tadpoles [5]. A simlar observation in juvenile rats was attributed to the presence of afferent fibers in the ventral roots (VRs) [6], a possibility that was subsequently ruled out [7, 8]. Recurrent excitation was also described in neonatal mice [9], in which VR stimulation elicited a small postsynaptic response in Mns.

None of the previous studies provided a comprehensive analysis of the extent of recurrent excitation, and they only illustrated a few recordings of small evoked currents. Furthermore, there are contrasting reports on the type of receptors mediating recurrent excitation, with evidence showing sensitivity to either glutamatergic antagonists [6], cholinergic antagonists [7], or both [9]. Here, we perform a systematic study of recurrent excitatory circuitry and demonstrate that recurrent excitation between Mns is strong and it is maintained throughout development into maturity. Our data show that while recurrent excitation between intrasegmental and intersegmental Mns is comparable in size, fast-type Mns receive a 10-fold greater amount of recurrent excitation compared to slow Mns. Under normal physiological conditions, recurrent excitation can override recurrent inhibition, and Mn firing in one spinal segment propagates to neighbouring segments. Remarkably, while acetylcholine and a mixture of acetylcholine and glutamate act at the neuromuscular junction and RC synapses, respectively [8, 9], neurotransmission between Mns is purely glutamatergic.

## Results

**Fig 1:**
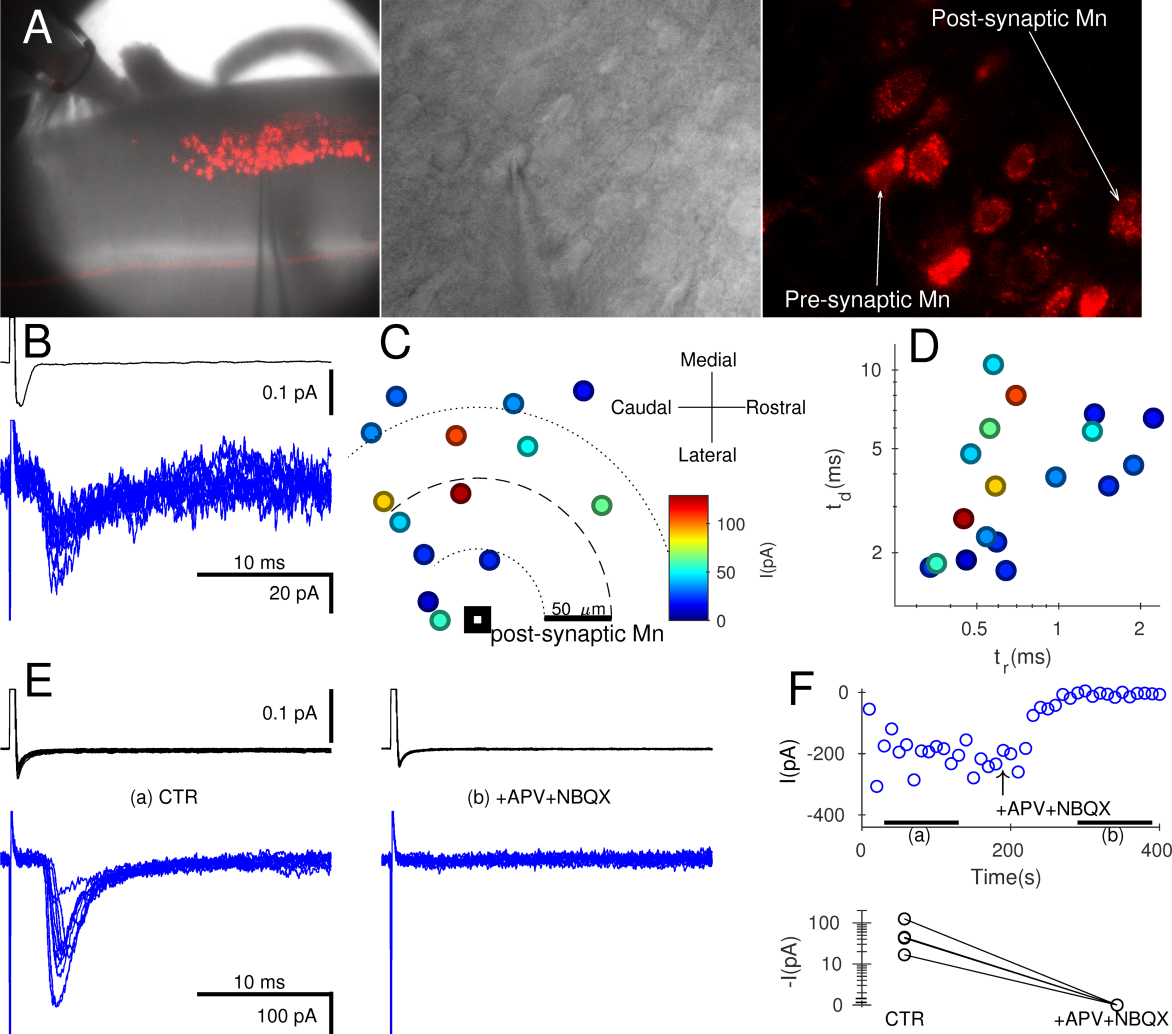
Paired recordings from Mns showed small unitary currents that were purely glutamatergic. Prior intramuscular injection of gastrocneumius with CTB-Alexa-Fluor-555 fluorescently labelled the dorsal motor column (A) within the coronal preparation (left) for simultaneous visualisation of Mns using infrared (middle) and confocal (right) optics. Presynaptic cells were stimulated in loose cell-attached voltage clamp (B, upper trace) while recording evoked postsynaptic responses in whole-cell voltage clamp (B, lower trace). Connected Mns were usually within 150 μM of one another, and in most paired recordings (14/18), their respective locations were recorded (group data tabulated in S1 Data). Graph C plots the relative position of each presynaptic Mn in relation to the postsynaptic cell, with colour-coded size of the corresponding reponses. A similar colour code and scale is used in graph D, showing decay time (t_d_) against the rise time (t_r_). Using oblique slice preparations (see text), we investigated the pharmacology of the synapse. Panel E shows a representative recording in control (left) and following glutamatergic blockade (E, right) using APV and NBQX. The time course of changes in evoked responses during the bath application of the antagonists showed complete suppression of currents (F, top). Similar effects were observed for all 4 paired recordings (F, bottom). APV, D-2-amino-5-phosphonopentanoic acid; CTR, control conditions; Mn, motoneurone; NBQX, 1,2,3,4-tetrahydrobenzo(f)quinoxaline-7-sulphonamide.

We performed paired recordings to measure the efficacy of unitary connections in fluorescently labelled Mns innervating gastrocnemius. Simultaneous infrared and confocal imaging was used to identify and patch fluorescent Mns in a dorsal horn–ablated spinal cord (Fig 1A). Strychnine (0.5 μM) and gabazine (3 μM) were applied to block recurrent inhibition. Mns were patched in whole-cell voltage clamp while putative presynaptic cells were stimulated in loose-cell attached configuration (see Materials and methods) until an evoked response was detected in the postsynaptic cell. Fig 1B shows an example of a paired recording with an average evoked current of −34 pA. A location map constructed from 14 out of the 18 recorded pairs (Fig 1C) shows that connected Mns tended to be within 150 μM from one another but with no systematic relationship between distance and size of response (range 11–125 pA).

The rise and decay times of evoked currents (Fig 1D, response size colour coded) were fast, with a median rise time of 0.59 ms and decay time of 3.75 ms. There was, however, no correlation between either of the kinetic parameters and the size of response. In oblique slice preparations (see Materials and methods), we assessed the pharmacology of evoked responses (Fig 1E). The postsynaptic current was fully abolished by bath application of 50 μM D-(-)-2-Amino-5-phosphonopentanoic acid (APV) and 2 μM 2,3-Dioxo-6-nitro-1,2,3,4-tetrahydrobenzo[*f*]quinoxaline-7-sulfonamide disodium salt (NBQX), to block N-Methyl-D-aspartate (NMDA) and α-amino-3-hydroxy-5-methyl-4-isoxazolepropionic acid (AMPA) receptors, respectively (Fig 1F, top). Identical results were obtained from all 4 pairs tested (Fig 1F, bottom).

**Fig 2:**
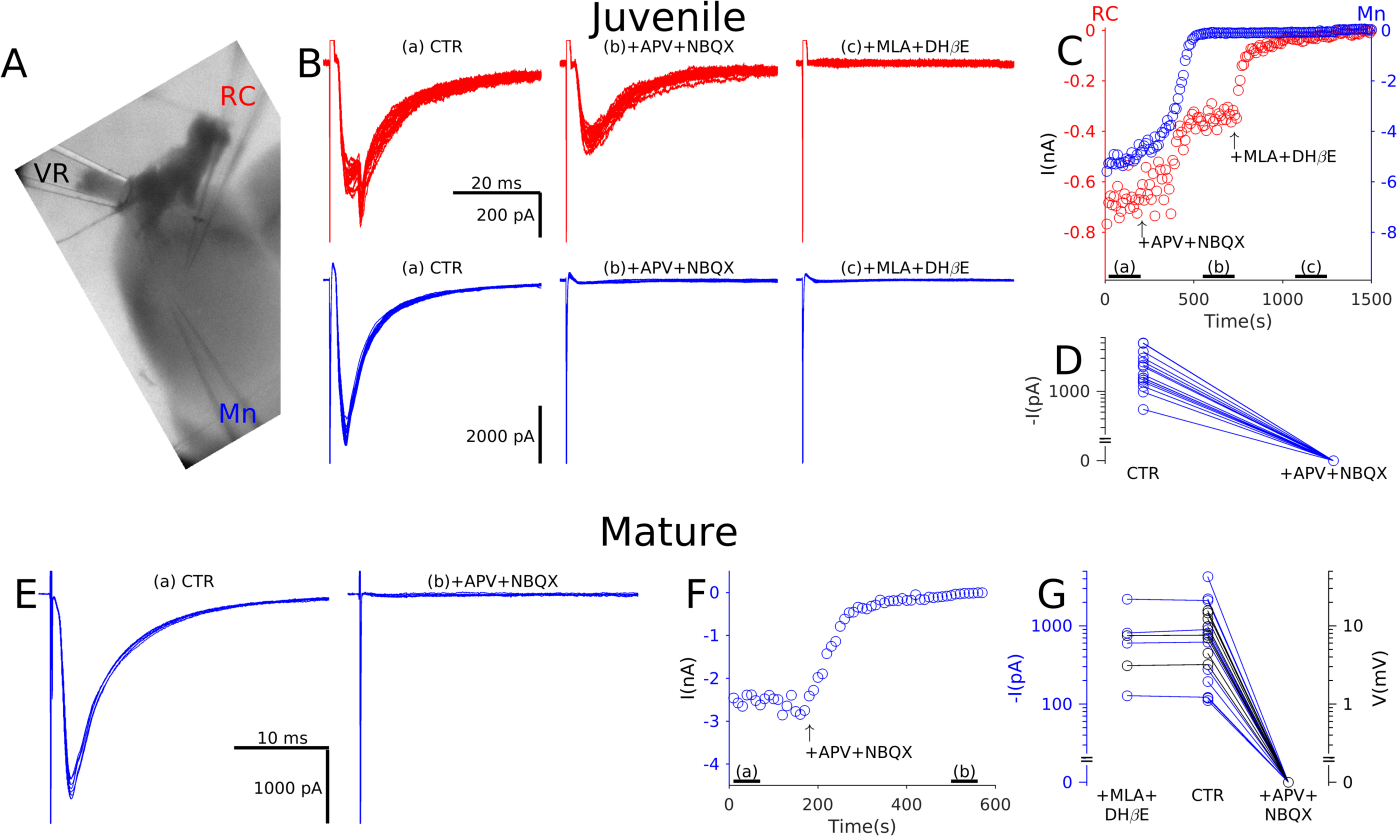
Electrophysiological recordings from both juvenile and mature preparations showed that recurrent excitation in Mns is purely glutamatergic. In oblique slice preparations (A) a suction electrode was used to stimulate the VR while recording respones from an Mn and, in the illustrated example, also from an RC. Note that the RC response shown includes a second component originating from a gap junction with a neighbouring RC. Panel B illustrates postsynaptic responses (stimulus artefacts truncated) of the RC (red, top) and Mn (blue, bottom) in control (left), in the presence of glutamatergic antagonists (middle) and following block of cholinergic transmission with MLA and DHβE (right). The time course of changes in currents (C, top) during application of the antagonists showed partial attenuation of RC responses and full suppression of Mn responses in the presence of glutamatergic antagonists. RC currents were only abolished by block of cholinergic receptors. In all Mns tested, the rEPSC was completely abolished by glutamatergic antagonists (D, group data tabulated in S1 Data). Similar experiments were performed on mature preparations in which Mn responses to VR stimulation were recorded in control (E, left) and during glutamatergic blockade (E, right). Once again, the time course of changes in reponses (F) showed complete suppression of Mn responses in the presence of glutamatergic antagonists. Graph G summarises the group data from Mns recorded in voltage clamp (black) and current clamp (blue) showing no effect following bath application of cholinergic antagonists, whereas glutamatergic blockade completely abolishes responses. APV, D-2-amino-5-phosphonopentanoic acid; CTR, control conditions; DHβE, dihydro-β-erythroidine; MLA, methyllycaconitine; Mn, motoneurone; RC, Renshaw cell; rEPSC, recurrent Excitatory Postsynaptic Current; VR, ventral root.

Because the tested unitary connections might have represented a specific local subset of the entire population of Mn–Mn synapses, we investigated the pharmacology of currents evoked by VR stimulation, thus pooling responses to all inputs from a given segment (Fig 2A). In the example of Fig 2A–2C, we simultaneously recorded from an RC (Fig 2B, top, red) and an Mn (Fig 2B, bottom, blue). The RC response shown includes a second component originating from a gap junction [10], contacting a neighbouring RC in which VR stimulation evoked an action potential.

Whereas bath application of glutamate antagonists resulted in a reduction of the RC response to approximately 50%, the response in the Mn is completely abolished. The remaining cholinergic component of the response in the RC was blocked by further application of 10 nM methyllycaconitine (MLA) and 5 μM dihydro-β-erythroidine (DHβE) to block α7 and αβ receptors, respectively (Fig 2C). Group data from 16 Mn recordings are illustrated in Fig 2D. The mean latency (±SEM) of responses of 1.60 ± 0.13 ms and the corresponding response jitter, quantified using the standard deviation of the latencies, was 0.08 ± 0.01 ms, consistent with monosynaptic responses to VR stimulation. In all cases, application of glutamate antagonists resulted in complete suppression of evoked currents (Fig 2D).

While the data from Fig 2D were obtained from juvenile mice (P7–14), we performed similar recordings from more mature animals (P15–25) to determine whether pure glutamatergic transmission is preserved throughout development. Fig 2E and 2F shows that the response is fully suppressed by glutamatergic blockade. In 20 Mns recorded in voltage clamp (black, Fig 2G) or in current clamp (blue, to reduce the duration of the stimulus artefact), glutamatergic antagonists entirely suppressed responses, whereas prior cholinergic blockade had no significant effect (*n* = 6, Wilcoxon sign-rank *z* = −0.53, *P* = 0.600). The mean latency of the responses was 1.54 ± 0.22 ms, with a jitter of 0.13 ± 0.03 ms.

**Fig 3:**
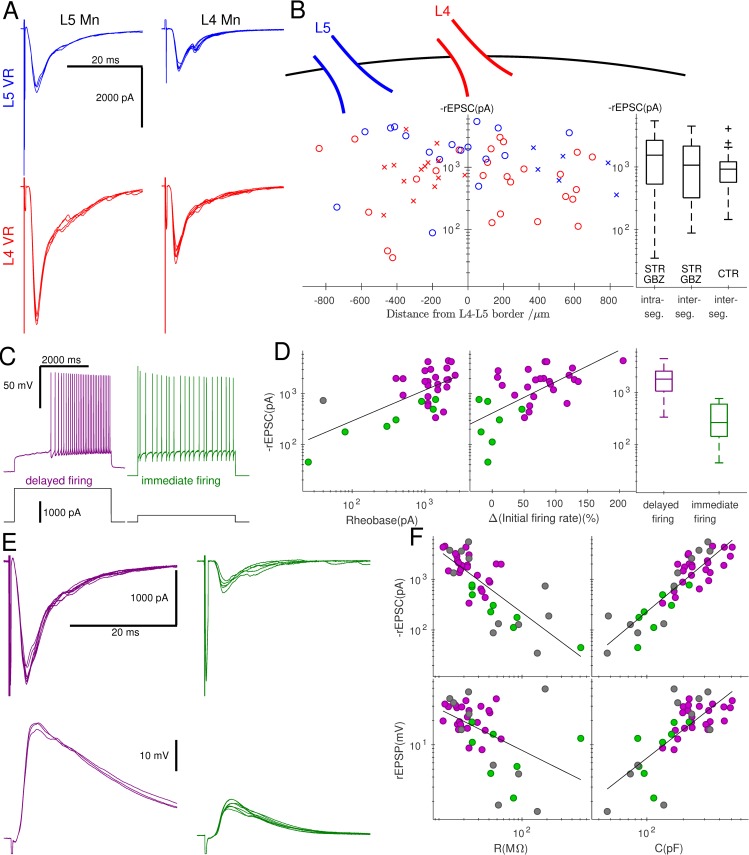
Recordings from coronal preparations showed that the magnitude of rEPSCS was related not to the position of the Mn but to its firing type. Example traces of evoked responses (stimulus artefacts truncated) of Mns located in L5 (A, left) and L4 (A, right) segments are illustrated following stimulation of the L5 (A, top, blue) and L4 (A, bottom, red) VR; the response in the top right trace exhibited a late component, suggesting activation of a disynaptic pathway. Panel B shows the size of rEPSCs recorded from all Mns (group data tabulated in S1 Data) against their distance from L4–L5 border (rostral positive) colour-coded according to whether L5 (blue) or L4 (red) VR was stimulated using crosses for control and circles in the presence of strychnine and gabazine. There was no systematic association between rEPSCs and position as shown in the box and whisker plot (B, right) comparing intrasegmental to intersegmental responses or when comparing intersegmental responses recorded in the presence of inhibitory antagonists with control conditions. Two Mn cell types were distinguished using current clamp recordings on the basis of whether at rheobase, positive current application elicited delayed (C, left, purple), or immediate (C, right, green) firing. Delayed-firing cells (purple) were associated with a high rheobase (D, left), an accelerating initial firing rate (D, middle), and large evoked rEPSCs (D, right) in comparison to immediate-firing cells (green). The traces in panel E illustrate representative responses to VR stimulation from a delayed-firing cell (purple, left) and immediate-firing cell (green, right) recorded in voltage clamp (top) and current clamp (bottom). Graph F shows the group data, plotting the rEPSCs and rEPSPs against cell resistance and capacitance, using grey circles to denote cells that were not identified by their firing pattern at rheobase. In all 4 cases, correlations were observed, demonstrating that responses were greater in larger Mns, which tended to be of the delayed-firing type. CTR, control conditions; GBZ, gabazine; Mn, motoneurone; rEPSC, recurrent excitatory postsynaptic current; rEPSP, recurrent excitatory postsynaptic potential; STR, strychnine; VR, ventral root.

We next investigated whether recurrent excitation could propagate across segments in coronal preparations (see Materials and methods) from juvenile mice (P7–14) in which Mns innervating gastrocnemius were labelled. Fig 3A illustrates recurrent excitatory postsynaptic currents (rEPSCs) recorded in L5 (left) and L4 (right) Mns while stimulating the L5 (upper, blue trace) or L4 (lower, red trace) VR. The rEPSC size from 43 recordings from L4 and L5 Mns is plotted against the distance from the L4/L5 border, colour coded to represent responses evoked by L4 (red circles) or L5 (blue circles) VR stimulation (Fig 3B). There were no obvious differences in rEPSC size between the 2 stimulated roots or between L4 and L5 Mns. Comparison of rEPSCs from responses to VR stimulation from the same segment or neighbouring segment showed no significant differences (Fig 3B right, Wilcoxon rank-sum *z* = 0.61, *P* = 0.541). Despite the lack of correlation between the size of the current and the position of the recorded Mn with respect to the stimulated VR, we observed a very broad distribution of amplitudes of rEPSCs, with sizes ranging from 60 to more than 5,000 pA (see S1 Fig, showing the rEPSCs recorded in all Mns in which the position was registered.)

In the experiments above, the rEPSCs were pharmacologically isolated by blocking fast inhibitory transmission with strychnine and gabazine, in order to avoid potential bias in the current measurements, due to the opening of the large inhibitory conductances associated with the activation of RCs following VR stimulation. However, because the cord might become hyperexcitable following full block of synaptic inhibition, we tested whether propagation of recurrent excitation across segments was preserved even with intact inhibition. In 3 different preparations, we stimulated either L4 or L5 VRs and recorded the rEPSCs from 21 Mns located in the adjacent L5 or L4 segment (see individual responses in S2 Fig). Amplitudes from experiments performed without block of synaptic inhibition are shown as red (descending) or blue (ascending) crosses in Fig 3B (left). The box and whisker plot (Fig 3B, right) shows that a comparison of intersegmental responses in the presence and the absence of antagonists of inhibition shows no significant differences in size (Wilcoxon rank-sum *z* = 0.48, *P* = 0.635).

The mean latency (±SEM) of responses of 1.64 ± 0.09 ms was consistent with monosynaptic activation and not significantly different from those from oblique slices (1.60 ± 0.13 ms, Wilcoxon rank-sum *z* = −0.90, *P* = 0.384). Latencies of responses from Mns in neighbouring segments (1.77 ± 0.13 ms) were longer compared to those recorded from within the same segment that was stimulated (1.43 ± 0.09 ms, Wilcoxon rank-sum *z* = −2.43, *P* = 0.015). The corresponding response jitters were very small both within (0.08 ± 0.03 ms) and across (0.08 ± 0.01 ms) segments, and a comparison between the two showed no significant difference (Wilcoxon rank-sum z=−0.58, *P* = 0.559). These results demonstrate that, while the latency of synaptic responses may be greater across segments compared to within segments, they are both mediated by monosynaptic connections between Mns within and across segments.

**Fig 4:**
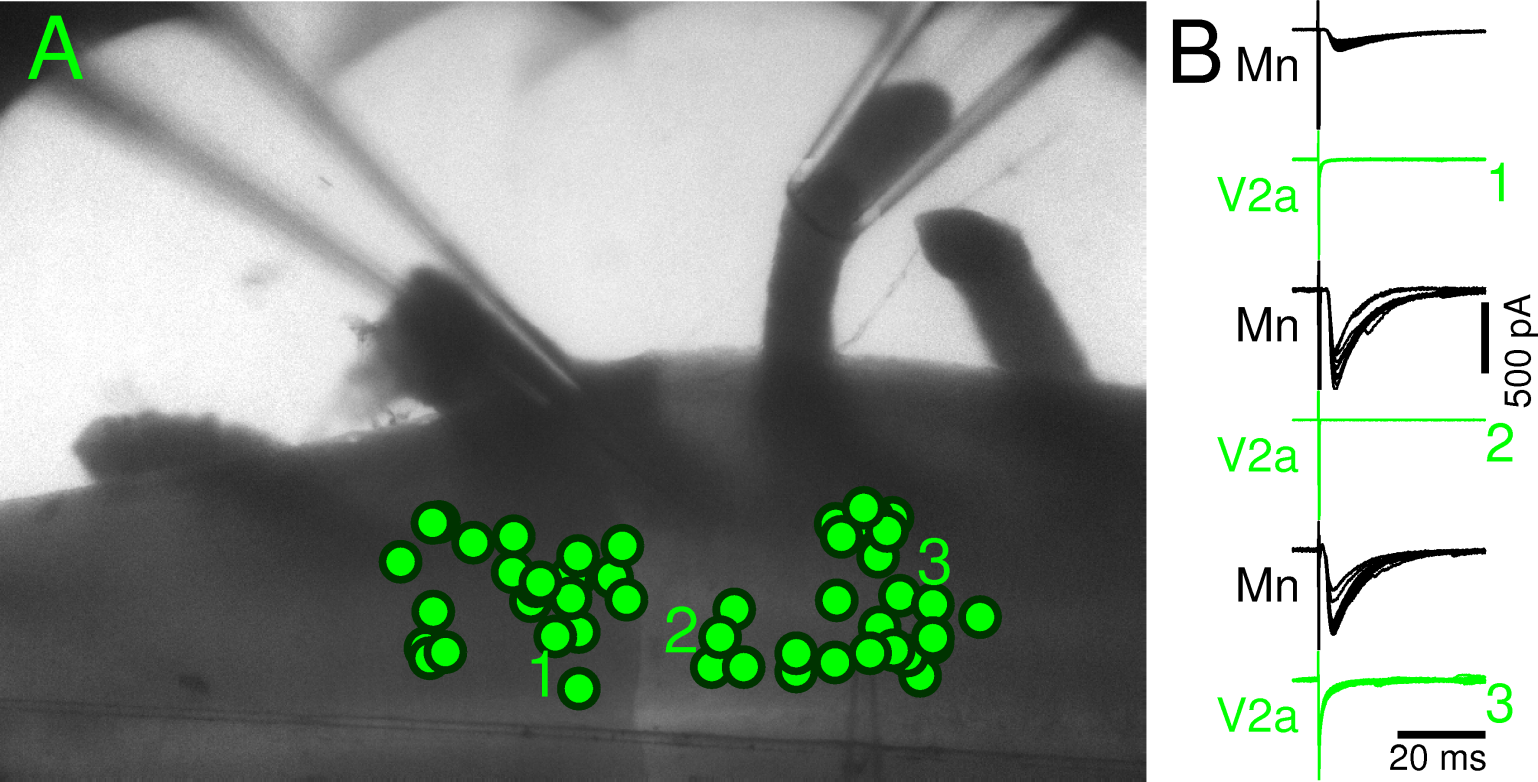
V2a interneurones do not respond to VR stimulation. Panel A shows the location of 42 recorded V2a interneurones, identified by the expression of EGFP. The cells labelled with numbers are shown in panel B and were recorded simultaneously with an Mn. Stimulation of a VR elicited a large current in Mns but no response in the recorded V2a interneurones. EGFP, enhanced green fluorescent protein; Mn, motoneurone; VR, ventral root.

It has been recently demonstrated that in zebrafish, Mns are electrically coupled with V2a interneurones that in turn project back to motor nuclei with glutamatergic synapses [11]. It is therefore possible that VR-evoked, synchronized antidromic spikes could elicit firing in V2a interneurones, which in turn could give rise to the observed short-latency, low-jitter EPSCs. In order to test this possibility, we used a dorsal horn–ablated preparation taken from mice selectively expressing enhanced green fluorescent protein (EGFP) in V2a interneurones. While stimulating a VR, we performed simultaneous whole-cell recordings from an Mn and cell-attached recordings from a V2a interneurone. At a stimulation intensity that elicited a maximal rEPSC in the Mn, no spikes could be evoked in the V2a interneurones in 3 different preparations (*n* = 46, V2a interneurones tested). We also performed simultaneous whole-cell recordings of Mns and V2a interneurones, and in *n* = 9 cells, we could not elicit any synaptic or electrically mediated current from the interneurone (one example of a double recording in each preparation is shown in Fig 4B) throughout the stimulated or adjacent segment of the spinal cord. The position of all recorded cells is overlayed with a picture of a cord in Fig 4A. These results therefore exclude any involvement of V2a interneurones in mediating the rEPSCs.

We next assessed whether the magnitude of recurrent excitation was related to the intrinsic properties of postsynaptic Mns. Two types of Mns were identified according to their firing pattern at rheobase in current clamp recordings [12]. The first type (Fig 3C, left, purple) has high rheobase, produces delayed firing with a pronounced increase in firing rate during positive current application, and is associated with fast-type units. By contrast, the second type (Fig 3C, right, green) has a lower rheobase and immediate firing with little change in spike frequency, characteristic of slow-type units [12]. High rheobase (Fig 3D, left) and accelerating initial firing (Fig 3D, middle) were correlated with the size of rEPSCs, with median values of 1,814 in the delayed-firing cells and 267 in the immediate-firing cells. Comparison between the 2 groups confirmed a significant difference (Fig 3D right, Wilcoxon rank-sum *z* = 3.72, *P*<0.001).

Differences between the 2 cell types are also associated with their passive properties, with delayed-firing Mns showing lower resistances (median 22 MΩ) and higher capacitances (median 237 pF) than their immediate-firing counterparts (median resistance 44 MΩ, median capacitance 125 pF), both at statistically significant levels (Wilcoxon rank-sum |z|≥2.94, P≤0.003). Recurrent excitatory responses were recorded from delayed-firing (Fig 3E, purple) and immediate-firing (Fig 3E, green) cells in both voltage clamp (top) and current clamp (bottom). Pooling all cell types together, correlations were observed between the size of response and resistance or capacitance (Spearman |*r*|≥0.516, *P*<0.001, Fig 3F).

**Fig 5:**
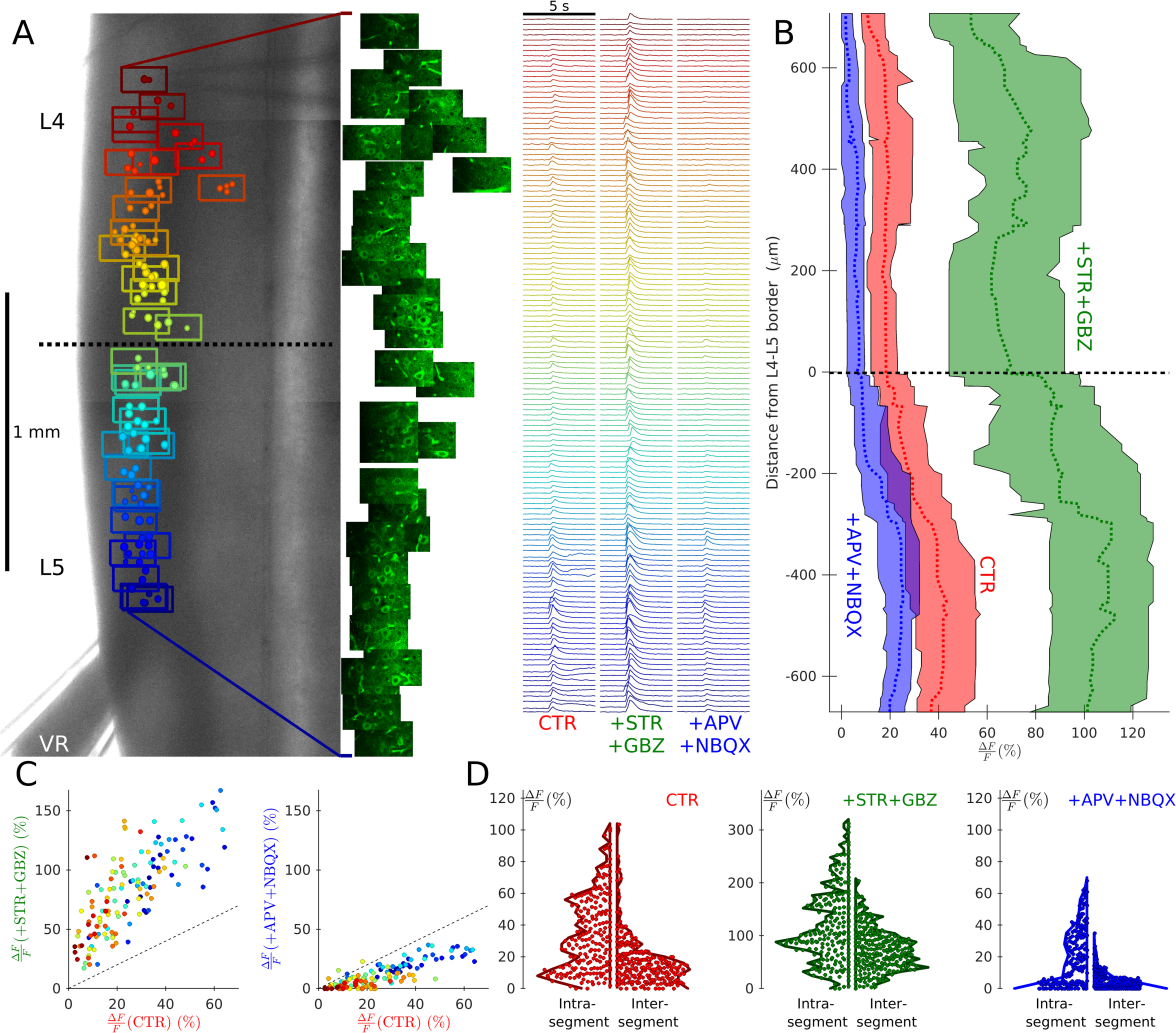
Calcium imaging from coronal preparations showed that recurrent excitation from one segment can evoke spikes in adjacent segments with intact recurrent inhibition, but these responses are abolished by glutamatergic antagonists. An example of such a recording is illustrated in (A), which overlays the Mn positions within optical fields colour coded by position, on top of a low-magnification image (A, left) of a coronal L4–5 preparation from a ChAT-GCaMP6s mouse in which Mns express GCaMP6s. A suction pipette was used to stimulate the L5 VR while acquiring fluorescence intensities throughout each field (A, middle). Changes in Mn fluorescence were measured and plotted against time (A, right) under CTR in the presence of pharmacological blockade of inhibition (+STR+GBZ) and addition of further antagonists to block glutamatergic neurotransmission (+APV+NBQX). The results for this experiment are summarised in graph B, in which running medians and interquartile ranges of responses are plotted for the 3 conditions against the distance from the L4–5 border (rostral positive). Recurrent excitation from L5 evoked firing in Mns throughout the L4 segment, and these responses were enhanced by inhibitory antagonists and abolished by glutamatergic blockade. These effects are illustrated by the graphs in (C), which plots the signal response under control conditions with that in the presence of antagonists of inhibition (C, left) and glutamatergic blockade (C, right), preserving the colour coding of position in the rostral–caudal axis used in panel A. Violin plots summarise the group data (tabulated in S1 Data), comparing intrasegmental and intersegmental responses, showing the distribution of the magnitude of responses from all Mns under control conditions (D, left), inhibitory blockade (D, middle; on a different *y*-scale), and in the additional presence of glutamatergic antagonists (D, right). APV, D-2-amino-5-phosphonopentanoic acid; ChAT, choline-acetyltransferase; CTR, control conditions; GBZ, gabazine; GCaMP6, Mn, motoneurone; NBQX, 1,2,3,4-tetrahydrobenzo(f)quinoxaline-7-sulphonamide; STR, strychnine; VR, ventral root.

The presence of strychnine and gabazine during electrophysiological recordings precluded evaluation of whether recurrent excitation could override recurrent inhibition. We therefore conducted calcium imaging experiments in mice selectively expressing GCaMP6s in Mns to evaluate the propagation of recurrent excitation across different segments with recurrent inhibition intact. Fig 5A–5C illustrates a coronal preparation with a suction electrode applied to the L5 VR (Fig 5A, left). Calcium signals were acquired throughout the dorsal motor column of L4 and L5 (Fig 5A, middle), with 146 ms frame interval before, during, and following a train of 3 VR stimulations at 30 Hz. The signal from regions of interest, defined within the outline of Mn somata, was evaluated for the period of acquisition under control conditions, in the presence of 0.5 μM strychnine and 3 μM gabazine, and following application of 50 μM APV and 2 μM NBQX (Fig 5A, right).

While the latency of the rEPSCs recorded electrophysiologically was short (1.64–1.77 ms) and exhibited a very low jitter (0.08 ms), a greater variability in the apparent onset of the calcium signals was observed (Fig 5A, middle). However, analysis of each individual trace confirmed that the latency of responses were always within 150 ms to 300 ms. Because the responses were evoked by a single trial of a train of 3 stimuli applied over a 100-ms period, and images were acquired at a frame interval of 146 ms using a calcium indicator with slow kinetics (approximately 200 ms [13]), a variable shift of 1 to 2 frames was expected.

In control, recurrent excitation evoked spikes in Mns from both L4 and L5 segments, as shown by the running medians and interquartile ranges of the relative fluorescence signal (Fig 5B, red) throughout both segments. Bath application of strychnine and gabazine resulted in substantial amplification of responses throughout the motor column (Fig 5B, green), whereas additional application of glutamatergic antagonists abolished responses from the L4 segment and substantially attenuated those from L5 Mns (Fig 5B, blue). The residual response in L5 Mns reflects antidromic activation. Scattergrams, colour-coded by regions, comparing control responses to those during application of inhibitory antagonists (Fig 5C, left) and additional glutamatergic blockade (Fig 5C, right), confirm that while responses were greater in the lumbar regions closer to the stimulated VR, the relative effects of block of recurrent inhibition—or excitation—were similar throughout L4 and L5.

Group data from 461 Mns from 7 preparations are shown in Fig 5D, comparing responses within and across segments for the 3 conditions. In control, responses were significantly greater within the stimulated segment compared to outside (Fig 5D, left, Wilcoxon rank-sum *z* = 8.50, *P*<0.001), and these difference were maintained after block of inhibition (Fig 5D, middle) and excitation (Fig 5D, right) (*z*≥7.23, *P*<0.001). Pooling Mns from both segments, blockade of inhibition consistently increased the signal (Wilcoxon sign-rank *z*−18.59, *P*<0.001), whereas a significant reduction in signal was observed following additional application of glutamatergic antagonists (*z* = 18.20, *P*<0.001). Residual firing was mostly confined to Mns within the stimulated segment through antidromic activation, thus confirming the purely glutamatergic nature of recurrent excitation.

## Discussion

Our experiments show that strong recurrent excitation between Mns is maintained throughout development, and fast-type Mns receive greater recurrent excitation than slow ones. We demonstrate that synaptic transmission between Mns is purely glutamatergic. While it could be argued that the observed small unitary postsynaptic responses (approximately 100 pA) would have little effect on the excitability of Mns whose somata are very large, VR stimulation evoked responses usually exceeding 1 nA, indicating extensive convergence of segmental Mn populations.

Very few studies have examined, in any detail, recurrent excitation between Mns. In both available electrophysiological studies [7, 9], the size of the recurrent EPSCs is not reported, even though the examples shown suggest a small size—of the order of 100 pA—corresponding to the lower bound of our observations. The previous studies were performed on young neonatal animals (P0–P4), while we obtained all our recordings starting from the second week of age. Therefore, our estimates of the size of recurrent excitation is not directly comparable to that reported by [9] and [7] because most of the increase in input conductance and dendritic arborization occurs between P2–P4 and P10–P13 [14]. In the only previous study performed on more mature animals (P10–P20 rats [6]), recurrent EPSPs were reported in the range of 3 to 15 mV, similar to our observations in current clamp. While the conduction velocity reported in this study (0.35–0.96 m/s) would point towards stimulation of unmyelinated fibers, the consistent observation of anti-dromically induced firing strongly suggests that Mn axons were indeed stimulated, and the slow conduction thus most likely reflects incomplete myelination at this developmental stage.

The variation in the magnitude of rEPSCs is associated with Mn classification into delayed and immediate-firing types, with larger responses systematically observed in the delayed-firing, low-resistance, and high-capacitance cells. Our results are therefore consistent with a structural connectivity in which the fast-type larger Mns receive stronger recurrent excitation compared to slow-type smaller cells. This pattern of connectivity suggests that recurrent excitation could play a role in sequential recruitment of fast-type units during motor tasks in which progressively increasing muscular forces are needed. Alternatively, recurrent excitation might represent a closed-loop amplification circuit that reinforces and increases the firing rate preferentially in fast-type Mns and thus rapidly increases muscle contraction strength when required. Distinguishing between these two possibilities would require a full characterization of presynaptic Mns in order to determine whether it is slow or fast Mns that are preferentially connected to fast Mns.

In neonatal animals, VR stimulation can induce fictive locomotion [8] and entrain the spontaneous rhythmic bursting induced by block of inhibition [15]. Furthermore, optogenetic activation or silencing of motor pools alters the frequency and phase of chemically induced fictive locomotion [16]. These effects cannot be explained solely by recurrent excitation and may provide evidence for Mn collaterals contacting unidentified interneurones [17]. A similar finding has been recently reported in zebrafish, for which gap junctions between Mns and V2a interneurones can alter the swimming pattern [11]. In our experiments however, an involvement of gap junctions between Mns and V2a interneurones seems unlikely because in none of our recordings could we detect spikes in identified V2a interneurones following VR stimulation.

It is possible, however, that Mns synapse onto other, so far unidentified interneurones that, in turn, could project back to Mns. While the existence of such a disynaptic pathway is possible, it would not account for the large, constant latency responses observed both within and across segments. Indeed, in our electrophysiological recordings during pharmacological blockade of recurrent inhibition, we often observed a late disynaptic component. At present, we cannot ascertain whether the disynaptic current results from premotor interneurons or from orthodromic activation of Mn pools that were not antidromically activated by VR stimulation. In either case, such recruitment implies the existence of a positive-feedback amplifying circuit whose tendency to reverberate may be suppressed by recurrent inhibition.

The recurrent excitation characterised in the present study includes predominantly a monosynaptic component, and this is evidenced by three observations. First, the connectivity between Mn pairs must have been monosynaptic. Second, the latency of responses within (1.43 ± 0.09 ms) and between (1.77 ± 0.13 ms) lumbar segments were within the time-scale of neurotransmission through only a single synapse. Finally, the response jitters within (0.08 ± 0.03 ms) and between (0.08 ± 0.01 ms) segments were very small and virtually identical. These observations are only consistent with a monosynaptic connectivity between Mns both within the same segment and across neighbouring segments. While the occurrence of synaptic projections between Mns crossing spinal segments may be regarded as unusual, it is perfectly compatible with the known rostrocaudal distribution of Mn dendritic trees, which may span over 1 millimetre in juvenile mice with little or no change into adulthood [14].

A glutamate receptor–dependent effect on Mn EPSPs evoked by VR stimulation has been reported previously [6], but it was attributed to afferent fibres within the root [18], a possibility now excluded by subsequent labelling studies [8]. A previous study has reported a purely cholinergic response to VR stimulation in a small proportion (2/9) of Mns [9]. Across all electrophysiological recordings of the present study, however, there was not a single instance of a cholinergic component. The origin of such a discrepancy may result from differences in maturity because in the previous study [9] neonatal mice (P0-P4) were used, while our experiments were performed on mice of at least partially weight-bearing age (P7–P25).

Neurotransmission between Mns is purely glutamatergic, yet following normal maturation, the neuromuscular junction is solely cholinergic [19] and synaptic transmission of recurrent collaterals onto RCs is mixed with both cholinergic and glutamatergic components [20]. This remarkable dissociation demonstrates a differentiation of neurotransmission systems on the basis of the different postsynaptic targets of Mns. However, the presence of vesicular glutamate transporters in Mn collateral terminals is still controversial. Immunohistochemistry and in situ hybridization studies have reported the expression of the vesicular glutamate transporter 2 (VGlut2) in some Mn terminals onto RCs that are either positive [9] or negative [21] for the vesicular acetylcholine transporter. These respective findings indicate either coexistence or segregration of cholinergic and glutamatergic transmission of Mns onto RCs.

Others, however, have not detected the presence of VGlut2—or any other vesicular glutamate transporter—in Mn terminals [8, 22]. It is possible that such discrepencies arise from undetectable albeit functional expression levels of VGlut2. Another possibility is the existence of an unidentified vesicular glutamate transporter [8, 22]. This hypothesis is supported by the presence of glutamate-releasing C-fibres in the dorsal horn that are nevertheless negative for all known vesicular glutamate transporters [23, 24]. Because many Mn terminals may contain more aspartate than glutamate [25], it has been proposed that the released neurotransmitter could be aspartate. However, aspartate alone cannot activate AMPA receptors that mediate responses of RCs [26] or of the Mns characterised in the present study. Glutamate therefore remains the most likely candidate.

## Materials and methods

### Ethics statement

All experiments were carried out in accordance with the Animal (Scientific Procedures) Act (Home Office, UK, 1986) and were approved by the UCL Ethical Committee, under project licence number 70/7621. Intramuscular injections were performed under inhaled isofluorane anaesthesia and by a surgical procedure supervised and approved by the Veterinary Surgeon named by the Home Office, UK. Before being euthanized, animals were administered terminal anaesthesia via intraperitoneal injection of a mixture of ketamine and xylazine (80 mg/Kg and 10 mg/Kg, respectively).

### Transgenic animals

Experiments were performed on preparations obtained from male or female mice bred using a C57BL/6J background. For electrophysiological experiments with simultaneous recordings from Mns and RCs, a transgenic strain—in which the EGFP is expressed under the control of the promotor of the neuronal glycine transporter GlyT-2 [27]—was used to label glycinergic interneurones. Simultaneous recordings from Mns and V2a interneurones were performed on mice expressing EGFP under the control of the Chx10 transcription factor [28].

### Spinal cord preparations

Following anaesthesia by intraperitoneal injection of a mixture of ketamine/xylazine (80 mg/kg and 10 mg/kg, respectively), both juvenile and mature mice were decapitated and the spinal cord dissected in normal icecold aCSF containing (in mM) 113 NaCl, 3 KCl, 25 NaHCO_3_, 1 NaH_2_PO_4_, 2 CaCl_2_, 2 MgCl_2_, and 11 D-glucose (same solution was used for recording). The spinal cord was then glued onto an agar block and affixed to the chamber of a vibrating slicer (HM 650V, Microm, ThermoFisher Scientific, UK). We used a slicing solution containing (in mM) 130 K-gluconate, 15 KCl, 0.05 EGTA, 20 HEPES, 25 D-glucose, 3 kynurenic acid, and ph 7.4 with NaOH [29]. For cutting oblique slices, the cord was glued to an agar block cut at a 45-degree angle, with the ventral side facing the direction of the blade [20].

For coronally sliced preparations in which the dorsal horns were ablated, the cord was glued horizontally with the ventral surface facing upwards. A blade was used to transect the cord at the L1–L2 boundary at an angle that allowed visualization of the exact position of the central canal under a dissection microscope. The vibratome blade was then aligned to the central canal, and the ventral portion of the cord was sliced away from the dorsal part. Alignment with the central canal was essential to ensure a consistent dorsoventral level of the ablation across different preparations and to retain the dorsal motor nuclei near the cut surface of the tissue.

Identical procedures were used for juvenile (P7–14) and mature (P15–25) animals. For older animals, we routinely cut the first slice within 8 minutes following decapitation. Because spinal cord preparations are extremely sensitive to anoxia, especially prior to slicing, we found that minimizing the time to obtain the first slice consistently resulted in viable preparations with healthy Mns [20].

### Electrophysiology

All recordings from postsynaptic Mns were performed with a Molecular Devices Axopatch 200B amplifier, filtered at 5 kHz and digitized at 50 kHz. Patch pipettes were pulled to resistances in the range of 0.8–2 MΩ when filled with (in mM) 125 K-gluconate, 6 KCl, 10 HEPES, 0.1 EGTA, 2 Mg-ATP, pH 7.3 with KOH, and osmolarity of 290 to 310 mOsm. During voltage clamp recordings, Mns were clamped at −60 mV with series resistances in the range of 2 to 10 MΩ compensated by 60% to 80%.

During paired recordings, loose cell–attached stimulation was used to evoke spikes in putative presynaptic Mns using an ELC-03X (NPI Instruments, Bauhofring, Germany) amplifier and a 4–5 MΩ pipette filled with normal aCSF [30]. Within each field of view (240 μm × 240 μm), typically up to 40 Mns could be visualized, but in order to avoid excessive mechanical disturbances of the tissue while recording from the postsynaptic target, only those located within the first 150 from the surface could be tested for connections. Typically, 1 Mn for every 40 tested was connected to the recorded cell.

VR stimulation was delivered to evoke rEPSCs in Mns using a glass suction electrode whose tip was cut to correspond with the size of the VR [31]. The stimulation intensity was increased until the size of the rEPSC remained constant, typically at 5× threshold. In order to exclude direct stimulation of the ventral white matter, VRs were only used if they were of sufficient length to afford no possible physical contact between the slice and suction pipette. This was tested before and after each recording by confirming that side-to-side movement of the suction pipette resulted only in movement of the root and not the slice. The position of the stimulating electrode for all the dorsal horn–ablated preparations is shown for the experiments performed in the presence and absence of block of synaptic inhibition in Supplementary S1 Fig and S2 Fig, respectively, and ranged between 200 μm and 1500 μm from the edge of the glass pipette to the point of entry of the VR.

For measuring the size of the excitatory response in some Mns, for which it was necessary to prevent action potentials, cells were hyperpolarised below their resting membrane potential. Measurements of synaptic current and potentials from these recordings were adjusted to their predicted value at −60 mV, assuming a reversal potential of 0 mV for excitatory conductances. All electrophysiological experiments were performed in the presence of 0.5 μM strychnine and 3 μM gabazine except where stated otherwise. Where indicated, excitatory receptors were blocked using APV, NBQX, MLA, or DHβE.

### Intramuscular injections

In order to label Mns innervating the ankle flexor gastrocnemius muscle, intramuscular injections were performed 2 to 5 days prior to recording. Inhalant isofluorane was used for the induction and maintenance of anaesthesia. Traction was applied to the lower limb, and an incision was made through the skin and deep fascia overlying the muscle. A Hamilton syringe loaded with a glass needle was used to inject 1 μl of CTB-Alexa-Fluor-555 (0.2% in 1× phosphate buffer saline) into the middle of the muscle belly over a period of at least 1 minute. The skin was closed by suture using a buried stitch before cessation of anaesthesia and recovery.

### Calcium imaging

Calcium imaging experiments were performed on animals selectively expressing the genetically encoded calcium indicator GCaMP6s in Mns. These mice were generated by crossing mice expressing Cre under the control of choline-acetyltransferase (ChAT-Cre; JAX mouse line number 006410) with animals with the gene expressing GCaMP6 flanked by a flox-Stop cassette (JAX mouse line number 028866 [32]). Upon recombination with Cre, GCaMP6 is selectively expressed in Mns and other cholinergic cells of the offspring. Because the only other population of ChAT-positive lumbar spinal cells are the cholinergic partition neurones located around the central canal, there was no ambiguity in the identification of Mns from their basal GCaMP6 fluorescence and position within the motor nuclei.

Dorsal horn–ablated coronal slice preparations (P9–12) were used for imaging experiments to visualise the dorsal motor nuclei in the L4 and L5 segments containing Mns innervating tibialis anterior, gastrocnemius, and peroneus longus close to the cut surface. A laser-scanning confocal unit (D-Eclipse C1, Nikon, UK) with a diode laser (λ=488 nm, power output from optic fibre 3–5 mW) was used to locate and record calcium signals from Mns reaching a depth of approximately 100 μm from the surface. Fields of 128 × 64 pixels (pixel size 1.38 μm and dwell 7.2 μs) were scanned with a frame interval of 146 ms over different regions throughout the dorsal motor column. Trains of 3 stimulii at 30 Hz were delivered to the VR (L4 or L5) while images were being acquired from at least 1 s before the onset of the first stimulus pulse. For each field, calcium signals were acquired for a total of 35 frames corresponding to approximately 5 s, and the position of each field was recorded.

Posthoc analysis was performed to quantify Mn responses. Within each field, single Mns were identified by their fluorescence, and regions of interests were defined by the contour profiles of their somata. The time course of excitation was measured using the change in mean fluoresence following stimulation divided by the baseline average. In some cases, slow drifts in fluorescence was corrected by fitting an exponential to the initial trace before the stimulus. Changes in fluorescence exceeding 2 standard deviations of the baseline noise were measured over a 1 s window following VR stimulation.

## Supporting information

S1 Data Group data for electrophysiological and optical recordings. (XLSX)

S1 Fig Individual responses in Mns following VR stimulations during block of inhibitory synapses. Details of the position of recorded Mns and stimulated VR are shown for 7 preparations. Position and corresponding response in individual Mns are colour coded for each preparation (2 to 9 Mns recorded in each cord). Stimulation artefacts are blanked, and the time of the stimulation is indicated by a coloured segment. Mn, motoneurone; VR, ventral root.
(TIF)

S2 Fig Individual responses in Mns followng VR stimulations with inhibition intact. The position of Mns and stimulated VRs is shown for 3 different preparations in which the rEPSCs were measured in the absence of any antagonists of synaptic inhibition. Responses in 3 to 7 Mns per preparation are shown. Blanked stimulation artefacts are indicated by a coloured line. Mn, motoneurone; rEPSC, recurrent excitatory postsynaptic current; VR, ventral root.
(TIF)
