# Supplementary figures and images for: Recurrent excitation between motoneurones propagates across segments and is purely glutamatergic
Source: PLoS Biol. 2018 Mar 14;16(3):e2003586. doi: 10.1371/journal.pbio.2003586 (PMC5851534; doi:10.1371/journal.pbio.2003586)

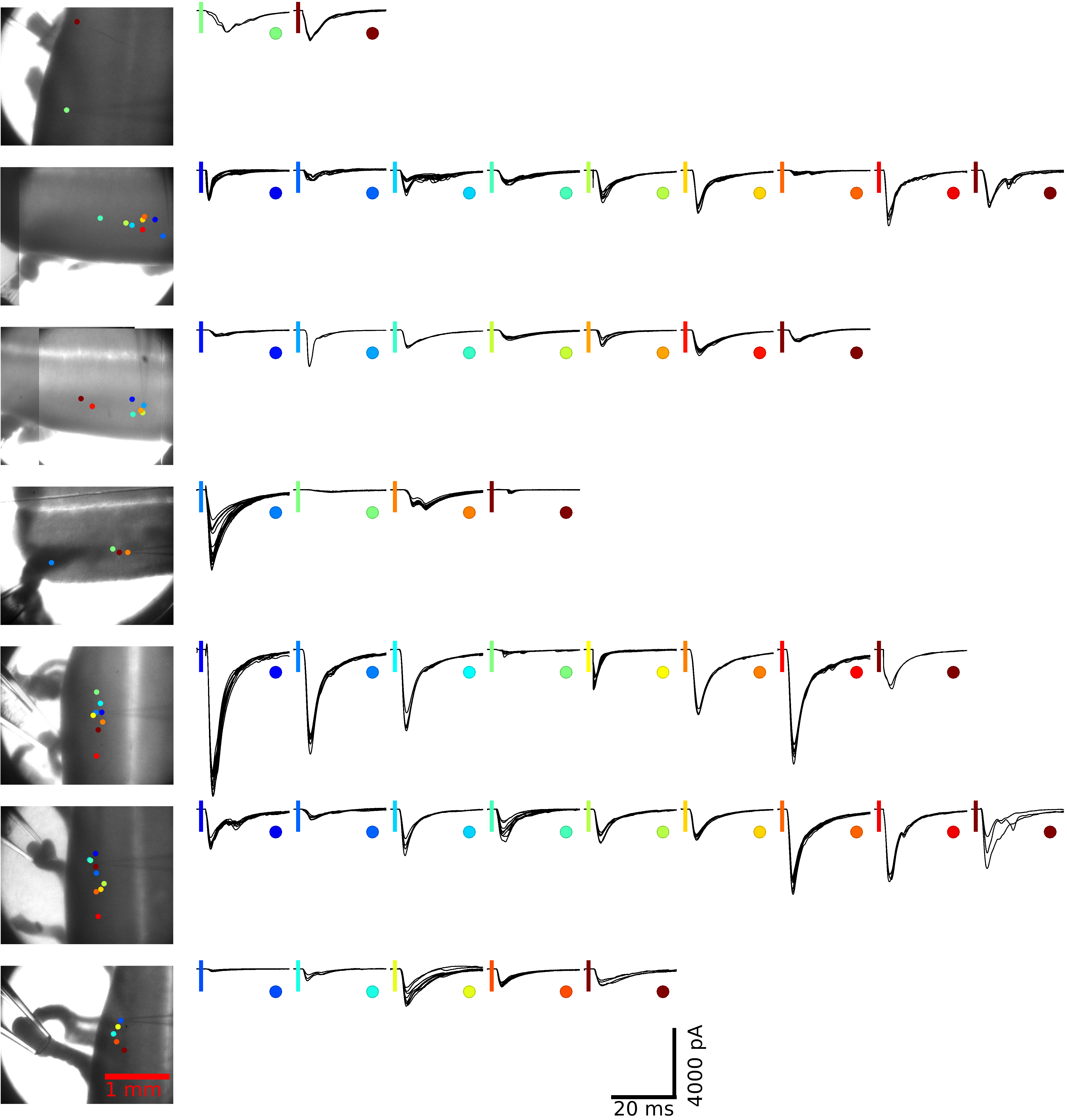

Supplement: S1 Fig — Details of the position of recorded Mns and stimulated VR are shown for 7 preparations. Position and corresponding response in individual Mns are colour coded for each preparation (2 to 9 Mns recorded in each cord). Stimulation artefacts are blanked, and the time of the stimulation is indicated by a coloured segment. Mn, motoneurone; VR, ventral root. (TIF) [file pbio.2003586.s002.tif]

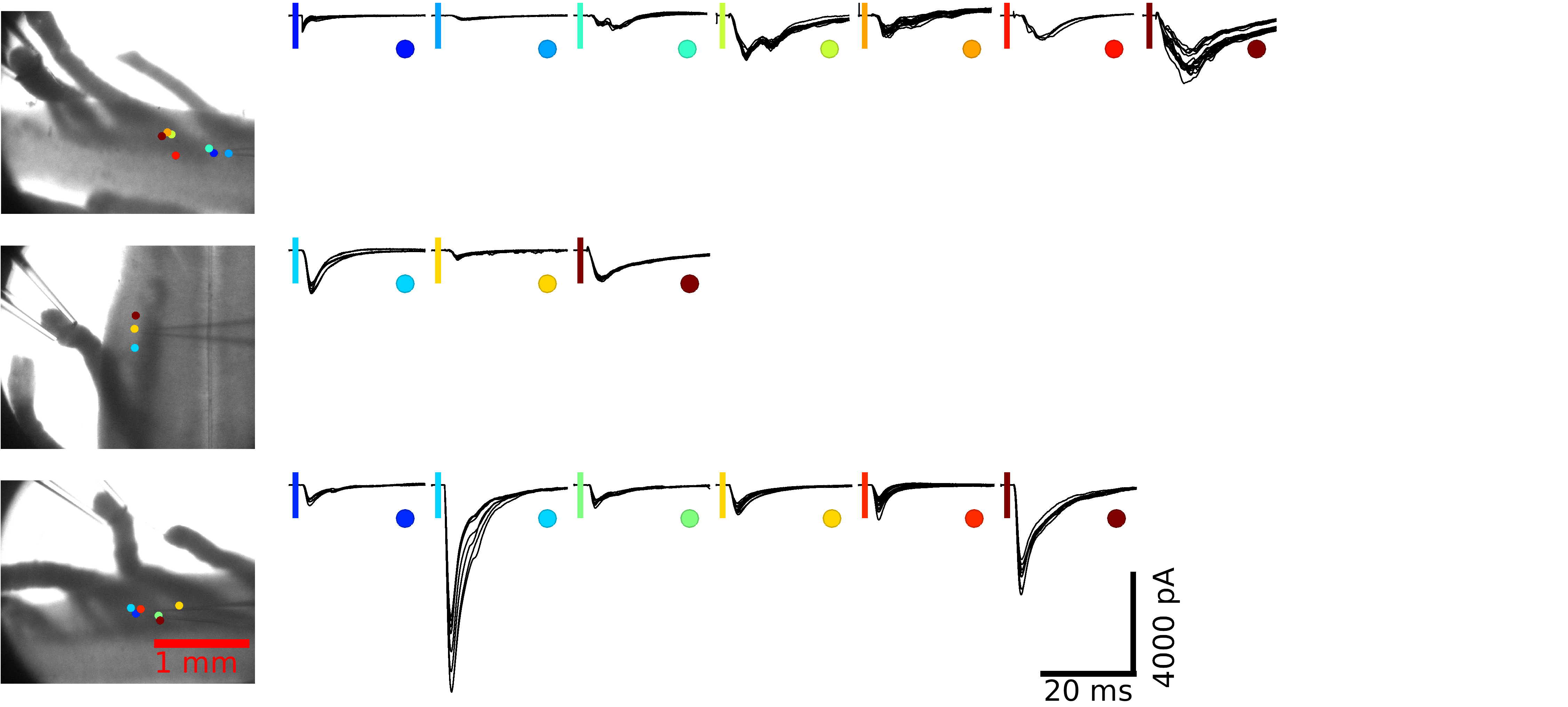

Supplement: S2 Fig — The position of Mns and stimulated VRs is shown for 3 different preparations in which the rEPSCs were measured in the absence of any antagonists of synaptic inhibition. Responses in 3 to 7 Mns per preparation are shown. Blanked stimulation artefacts are indicated by a coloured line. Mn, motoneurone; rEPSC, recurrent excitatory postsynaptic current; VR, ventral root. (TIF) [file pbio.2003586.s003.tif]
